# Examining the spatiotemporal evolution of vaccine refusal: nonmedical exemptions from vaccination in California, 2000–2013

**DOI:** 10.1186/s12889-018-5368-y

**Published:** 2018-04-24

**Authors:** Paul L. Delamater, Timothy F. Leslie, Y. Tony Yang

**Affiliations:** 1 Department of Geography, Carolina Population Center, University of North Carolina at Chapel Hill, Chapel Hill, NC USA; 2 Department of Geography and Geoinformation Science, George Mason University, Fairfax, VA USA; 3 Department of Health Administration and Policy, George Mason University, Fairfax, VA USA

**Keywords:** Vaccines, Vaccine hesitancy, Vaccine refusal, Exemptions, Geographic clustering

## Abstract

**Background:**

Vaccine hesitancy continues to be an issue throughout the United States, as numerous vaccine hesitant parents are choosing to exempt their children from school-entry vaccination requirements for nonmedical reasons, despite the safety and effectiveness of vaccines. We conduct an analysis of how vaccine refusal, measured by the use of nonmedical exemptions (based on personal or religious beliefs) from vaccination (NMEs), evolved across space and over time in California.

**Methods:**

Using school-entry data from the California Department of Public Health, we examined NMEs for students entering kindergarten in California from 2000 to 2013. We conduct global and local spatial autocorrelation analysis to determine whether NME use became more geographically clustered over the study period and whether the location of local clusters of high use were temporally stable. We conducted a grouping analysis that identified the general temporal trends in NME use over the time period.

**Results:**

The use of NMEs increased from 0.73% of all kindergarteners in 2000 to 3.09% in 2013 and became more geographically clustered over the study period. Local geographic clusters of high use were relatively isolated early in the study period, but expanded in size over time. The grouping analysis showed that regions with high NME use early in the study period were generally few (15% of all US Census tracts) and relatively isolated. Regions that had low initial NME use and moderate to large increases over the study period were located in close proximity to the initial high use regions. The grouping analysis also showed that roughly half of all tracts had 0% or very low NME use throughout the study period.

**Conclusions:**

We found an observable spatial structure to vaccine refusal and NME use over time, which appeared to be a self-reinforcing process, as well as a spatially diffusive process. Importantly, we found evidence that use of NMEs in the initially isolated regions appeared to stimulate vaccine refusal in geographically proximal regions. Thus, our results suggest that efforts aimed at decreasing future NME use may be most effective if they target regions where NME use is already high, as well as the nearby regions.

**Electronic supplementary material:**

The online version of this article (10.1186/s12889-018-5368-y) contains supplementary material, which is available to authorized users.

## Background

Despite the proven success of childhood vaccination programs in reducing or eliminating numerous vaccine-preventable diseases (VPDs), vaccine hesitancy continues to be an issue throughout the United States. Vaccine hesitancy is a delay in acceptance or refusal of vaccines despite their availability and is well-understood to be driven by a complex set of factors that include social norms, previous experiences, and personal beliefs among others [1]. While vaccine hesitancy is not a new phenomenon [2] in the US, recent increases in the number of parents that refuse vaccination for their children [3], as well as recent outbreaks of VPDs such as pertussis and measles [4, 5], have resulted a renewed interest in understanding vaccine-related behavior. Moreover, this phenomenon is not constrained to the US; numerous countries around the world are also contending with issues stemming from vaccine hesitancy, including reduced levels of coverage and corresponding VPD outbreaks [6, 7].

In the US, each state and the District of Columbia^1^ develops and implements their own childhood vaccination laws, regulations, and procedures, which are generally enforced at school entry [8]. For children with a contraindication for vaccination, all states have a provision for a medical exemption [9]. Further, nearly all states allow parents to obtain a nonmedical exemption (NME) for their child based on personal, philosophical, or religious beliefs. Currently, only California, Mississippi, and West Virginia do not provide this option. In states having an NME provision, the reasons for which they can be granted vary (e.g., personal and/or religious beliefs), as does the relative ease in which they can be procured. The restrictiveness of state-level NME requirements has been shown to affect the number of parents exempting their children from vaccination, as states with more restrictive policies tend to have fewer NMEs [10, 11]. Vaccine hesitancy in the US, as expressed through the use of NMEs, remains a highly contested topic across the country. In recent years, more than half of all states have considered and/or passed legislation that modified their existing NME requirements, including efforts to make NMEs both easier and more difficult to acquire [3, 9, 12].

Much of the research regarding vaccine refusal and NMEs has focused on understanding *who* are using NMEs, finding that parents choosing to acquire NMEs for their children tend to be well educated, high-income, and white, and that schools having high NME rates tend to be located in neighborhoods having similar characteristics [13]. Another active area of research has been to examine *where* parents choosing to use NMEs reside and whether they cluster geographically [e.g., 14–16]. Understanding where and why geographic clusters of low vaccination coverage have formed is important, as these regions can be at risk of losing herd immunity and outbreaks of VPDs [17–19]. Herd immunity is the indirect protection provided to those without immunity when overall vaccination coverage in the population is high, resulting in a decreased risk of disease transmission within the population [20]. Although children with NMEs cannot be assumed to be fully unvaccinated (given limitations of some surveillance systems) [21], numerous studies have shown a link between NME use and VPD outbreak risk [e.g., 14,22–24].

While the use of NMEs is usually associated with the refusal of one or more vaccines, vaccine hesitancy includes a range of potential parental behaviors regarding vaccines, from having or expressing concerns about vaccination (without action), to delaying the recommended schedule, to outright refusal [25]. It is generally accepted that those that refuse vaccines make up a very small proportion of all vaccine-hesitant parents [26]. Yet, the ability of those opposed to vaccination to disseminate their message and engage with other vaccine hesitant parents has been bolstered by the increasing availability and use of the Internet [2]. One particular concern is that parents will advance along the spectrum of hesitancy (e.g., from resistant to refusal) as the debate surrounding vaccines, vaccine safety, and parental rights continues to play out online [27].

Understanding vaccine hesitancy and VPD outbreaks in the US and other countries is and has been an active area of scientific inquiry; however, there has been less emphasis placed on how vaccine-related behavior changes over time and across space. There has been limited research on how vaccine refusal manifests throughout a region and whether there are observable spatial patterns that can shed light on the processes that drive hesitant or resistant parents to become vaccine refusers. The goal of this research is to initiate such an analysis by examining vaccine refusal over time via an analysis of the changing spatial patterns of NME use in a large study region. Evaluating NMEs for children entering kindergarten from year to year presents an interesting opportunity to understand this phenomenon, as there should be a different group of parents sending children to school each year.

We conduct an analysis in the state of California, where the NME rate for incoming kindergarteners increased from less than 0.5% in 1996–97 to more than 3% in 2013–14^2^ [28]. As a result of the increasing use of NMEs and the corresponding decrease in vaccination coverage within the state’s school system, California passed and implemented two laws aimed at curbing the use of NMEs. AB2109 was implemented prior to the 2014 school year and made NMEs more difficult to acquire by requiring all parents to receive counseling from a health care provider prior to obtaining a valid NME. SB277 was implemented prior to the 2016 school year and removed the NME provision entirely. We constrained our analysis to only consider the time period from 2000, the earliest year with publically available data, to 2013, the final school year prior to the implementation of AB2109. By restricting the analysis to this particular time period, we are able to examine the changes in vaccine-related behavior that were unaffected by the large-scale policy changes limiting California parents’ ability to acquire NMEs for their children.

We already know that state-level NME use increased in California from 2000 to 2013. Others have examined spatial clustering of NMEs in California over recent time periods in relation to pertussis outbreaks [14], school characteristics and medical exemptions [15], and policy changes [29]. This research is solely focused on the local spatial patterns of NME use and how they evolved over time. For example, we are interested in establishing whether the statewide increase in NME uses was largely driven by smaller increases distributed across the entire state or spatially isolated pockets of substantive change. We address the following research questions:

1. Did NME use become more spatially clustered over time?
2. Did the location of local spatial clusters of NME use change over time?
3. What were the general temporal patterns of NME use?

By examining the spatiotemporal nature of the changes in NME use, we aim to uncover whether vaccine refusal acted as a spatially diffusive or contagious process over this time period, as suggested by others [15, 29]. The first two research questions focus on the changing spatial patterns of vaccine-related behavior in an effort to unravel the role that “space” or “location” may play over time. In particular, we are interested in whether NME use in California demonstrated some form of spatial structure, which would shed light on the role of local behavior, e.g., spatial diffusion, wherein NME use within one region may stimulate hesitant parents in nearby regions to become vaccine refusers. The third research question places an emphasis on the temporal trends of NME use; this question explores the statewide increase in NME use as a function of various local-level changes over the study period.

## Methods

### Data and preprocessing

We acquired enrollment and vaccination data for kindergarteners entering California schools from 2000 to 2013 from the California Department of Public Health (CDPH, http://www.cdph.ca.gov). The database contains the total number of students entering kindergarten and the number of students with NMEs (among other vaccine-related information) for all public and private schools in the state with 10 or more kindergarteners enrolled. The yearly exemption and enrollment data were linked together via a unique school identifier code, and the school locations were geocoded per the method described in [30]. Table 1 contains the number of schools that submitted vaccination data to the CDPH and their kindergarten enrollment, the number represented in the school-level vaccination database (schools with 10 or more kindergarteners that reported information), and the number that were successfully geocoded for each year of data.

**Table 1:** Number of schools (SCH) and kindergarten enrollment (ENR) for schools reporting vaccine information, 2000–2013

	*Reported*		*Represented*				*Geocoded*			
YEAR	SCH	ENR	SCH	SCH(%)	ENR	ENR(%)	SCH	SCH(%)	ENR	ENR(%)
2000	8473	526,466	7418	87.55	521,198	99	7244	85.5	516,801	98.16
2001	8705	523,516	7502	86.18	517,854	98.92	7394	84.94	515,859	98.54
2002	8646	519,397	7428	85.91	513,560	98.88	7338	84.87	511,889	98.55
2003	8544	513,519	7352	86.05	507,680	98.86	7256	84.93	505,961	98.53
2004	8510	510,074	7361	86.5	504,450	98.9	7309	85.89	503,462	98.7
2005	8496	512,733	7371	86.76	507,224	98.93	7323	86.19	506,020	98.69
2006	8481	503,160	7364	86.83	497,817	98.94	7295	86.02	496,580	98.69
2007	8481	499,301	7309	86.18	493,626	98.86	7231	85.26	491,765	98.49
2008	8219	501,046	7173	87.27	496,027	99	7133	86.79	495,313	98.86
2009	8213	507,191	7124	86.74	491,653	96.94	7095	86.39	490,908	96.79
2010	8189	509,849	7163	87.47	505,015	99.05	7144	87.24	504,585	98.97
2011	8301	529,400	7236	87.17	524,336	99.04	7220	86.98	523,969	98.97
2012	8220	530,418	7192	87.49	525,536	99.08	7176	87.3	525,153	99.01
2013	7684	533,680	6982	90.86	530,530	99.41	6982	90.86	530,530	99.41

The Reported columns represent values for all schools that submitted data. The Represented columns contain values for schools represented in the vaccine database (i.e., schools with 10 or more incoming kindergarteners) and the Geocoded columns contain values for schools that were successfully geocoded. The percent values (%) in Represented and Geocoded refer to the percent of Reported schools and kindergarteners (e.g., in 2000, 85.5% of reporting schools and 98.16% of reported students are included in the final Geocoded dataset)

The yearly school-level data were spatially joined to their corresponding US Census Block Group (BG), US Census Tract, and School District (SD) per the methods in [31]. For the BG, Tract, and SD spatial data files, the enrollment and nonmedical exemption data for schools falling within each spatial unit were summed. For each level of data aggregation (schools, BGs, Tracts, and SDs) and for each year, the percent of kindergarteners with nonmedical exemptions (NME rate) was calculated by dividing the number of kindergarteners with an NME by the total number of kindergarteners. We also calculated year-to-year change in NME rate (raw percent) by subtracting the previous year’s NME rate, over all years in the dataset for each level of aggregation. Due to the data censoring of schools having less than incoming 10 kindergarteners, as well as school openings and closures occurring during the study period, not all observations had a full time-series record over the study period. Table 2 includes a breakdown of the number of observations by the number of years with data, over each level of data aggregation.

**Table 2:** Number and percent of observations disaggregated by years with enrollment and NME data

Years	School(n)	School(%)	BG(n)	BG(%)	Tract(n)	Tract(%)	SD(n)	SD(%)
1	653	6.84	267	3.51	111	1.99	11	1.34
2	411	4.31	198	2.60	69	1.24	7	0.85
3	321	3.36	151	1.98	68	1.22	5	0.61
4	323	3.38	164	2.15	64	1.15	4	0.49
5	250	2.62	148	1.94	70	1.26	2	0.24
6	268	2.81	141	1.85	79	1.42	5	0.61
7	241	2.53	133	1.75	61	1.09	5	0.61
8	278	2.91	163	2.14	83	1.49	5	0.61
9	289	3.03	201	2.64	100	1.79	4	0.49
10	280	2.93	202	2.65	111	1.99	5	0.61
11	237	2.48	185	2.43	100	1.79	6	0.73
12	297	3.11	234	3.07	142	2.55	11	1.34
13	695	7.28	533	7.00	340	6.10	49	5.98
14	5000	52.39	4892	64.27	4177	74.92	701	85.49
All	9543	100	7612	100	5575	100	820	100

Values are for Schools, Block Groups (BG), Tracts, and School Districts (SD). The row labeled All contains the total number of observations with at least one year of data within the study period (e.g., over 2000–2013, 9543 distinct schools reported at least one year of enrollment and exemption data). The number of observations (n) values represent counts (e.g., 653 schools reported only one year of data during the study period). The percent values represent the percent of All observations for that level of aggregation (e.g., 4.31% of the 9543 schools reported data for only two years of the study period)

### Neighborhood definition

Tests of spatial pattern require the definition of neighboring observations, and the process of defining these spatial neighborhoods can be difficult, given that limited theoretical or data-driven approaches offer a compelling justification for choosing one definition over another. In this work, we implemented a data-driven approach that leveraged Moran’s *I*, one of the most often used metrics to describe spatial autocorrelation [32], over multiple neighborhood definitions. We calculated the Moran’s *I* value for each year’s NME rate data, for each level of data aggregation. For the school-level data (point data), we used eight neighborhood definitions: Inverse Distance (ID) with distance thresholds of schools falling within 5, 10, 15, and 20 km of each observation and K Nearest Neighbors (KNN), using the 5, 10, 15, and 20 nearest schools for each observation. For the BG, Tract, and SD data (polygon data), we used the aforementioned eight neighborhood definitions, but also included first order contiguity neighbors using both queen and rook contiguity [33]. In each neighborhood definition, the neighbors were row standardized to account for the dissimilar number of neighbors (among observations) produced by some of the neighborhood definitions and to ensure consistency among the different neighborhood sets.

For each of the four levels of data aggregation, this approach produced 14 Moran’s *I* values for each neighborhood definition, corresponding to the 14 years of NME data (Additional file 1: Tables S1-S4). To choose which neighborhood definition to implement in our subsequent analysis, we used Pearson’s *R* to determine which of the neighborhood definitions produced results that were the most similar to the other definitions, on average. For each level of data aggregation, we constructed a correlation matrix containing the correlation between the Moran’s *I* values for the 14 years of data among the different neighborhood definitions, e.g., the correlation between the values of the school-level data using KNN(5) and ID(5) was 0.964. We then calculated the average correlation for each neighborhood definition, for each level of aggregation (Additional file 1: Table S5-S8). This process resulted in the selection of KNN(5) as the neighborhood definition for each level of data aggregation. The average *R* for KNN(5) was 0.966 (school-level), 0.954 (BG), 0.98 (Tract), and 0.864 (SD). Our approach can be summarized as follows: KNN(5) was the most similar to the other neighborhood definitions overall, and because of the high level of correlation with the other definitions, the results of the spatial analysis are not likely to be highly sensitive to alternate neighborhood definitions.

### Global spatial autocorrelation

Using the NME rate and NME rate change data, we calculated the global spatial autocorrelation for each year and each level of data aggregation using Moran’s *I* and Getis and Ord’s *G* [32]. Because global level autocorrelation metrics evaluate the spatial pattern of the entire set of observations simultaneously, these metrics allowed us to examine how the state-level spatial distribution of NME use changed over time. Moran’s *I* provides information on whether the values are clustered in space, randomly arranged, or dispersed, while Getis and Ord’s *G* provides information on whether the spatial clusters are more heavily influenced by clusters of high or low values. For all tests, we used KNN(5) for the neighborhood definition. The global autocorrelation analysis allowed us to evaluate whether or not NME use became more spatially clustered over this time period, as well as whether high or low NME use were more clustered.

### Local spatial autocorrelation

Using the NME rate data for each year and over each level of data aggregation, we evaluated local spatial autocorrelation and clustering using the Local Indicator of Spatial Association [LISA, 34]. This method identifies spatial clusters of high and low values, as well as high or low outliers (i.e., an observation with a high value that has neighbors with low values and vice versa). Preliminary analysis showed that, given the large number of observations with 0% NME rate, the algorithm did not detect clusters of low values. Thus, we also implemented a manual spatial lag analysis that identified regions that had a 0% NME rate and a complete set of neighbors with a 0% NME rate as local clusters of “low” values. For both the LISA and the spatial lag analyses, we used the KNN(5) neighborhood definition. The yearly LISA results were summed and converted to percent values, representing the percent of years that each observation was in a “High” cluster of NME rate values (high-high cluster or a high outlier). The spatial lag results of 0% NME rate were consolidated in a similar fashion, identifying the percent of years that each observation was in a “0%” cluster. The percent representation of years in a High or 0% cluster was reported in lieu of the number of years, as all observations did not have a full time-series record over the 14 years of data. The local spatial autocorrelation analysis allowed us to identify which regions of the state were consistently located in spatial clusters having high and low NME rates over the study period, as well as to visually identify spatial patterns in the results.

### Grouping analysis

To evaluate the general temporal patterns of NME use, we first needed to group the observations based on similar patterns of NME use over the study period. For each level of data aggregation, we grouped the individual observation units based on similarity across their yearly NME rates from 2000 to 2013; hence, observations placed in the same group exhibited similar patterns of NME use across the entire time period. The grouping analysis was performed separately for each level of data aggregation. Because this operation required observations to have a complete set of attribute values, the data were subset to include only those with NME rate data for all 14 years. We implemented the grouping approach in [35], which is summarized below. The approach uses the K-means clustering algorithm with seed locations provided by a Wards clustering algorithm to group the observations based on similarity across multiple characteristics, which were the yearly NME rate values. The grouping process was iterated over numerous values of *k*, the number of output groups. For this analysis, we evaluated solutions having between 2 and 30 groups. The incremental *F* score was used to identify which solutions (*k*) provide a large increase in explanatory power by allowing for an additional group (e.g., the gain in fit from increasing the number of output groups from 5 to 6). For this analysis, our main interest was evaluating the overall temporal patterns of the output groups (how NME rates changed over time), which required a relatively small number of output groups for interpretive purposes. Thus, to choose the number of groups for each level of data aggregation, we chose the solution (*k*) from the set 2–30 having the minimum number of groups that was also identified as a peak in the incremental *F* score analysis, per [35].

### Software

We used ArcGIS v10.3.1 [36] for basic mapping and data aggregation tasks. We used R v3.2.4 [37] for spatial analysis and grouping.

## Results

**Fig. 1:**
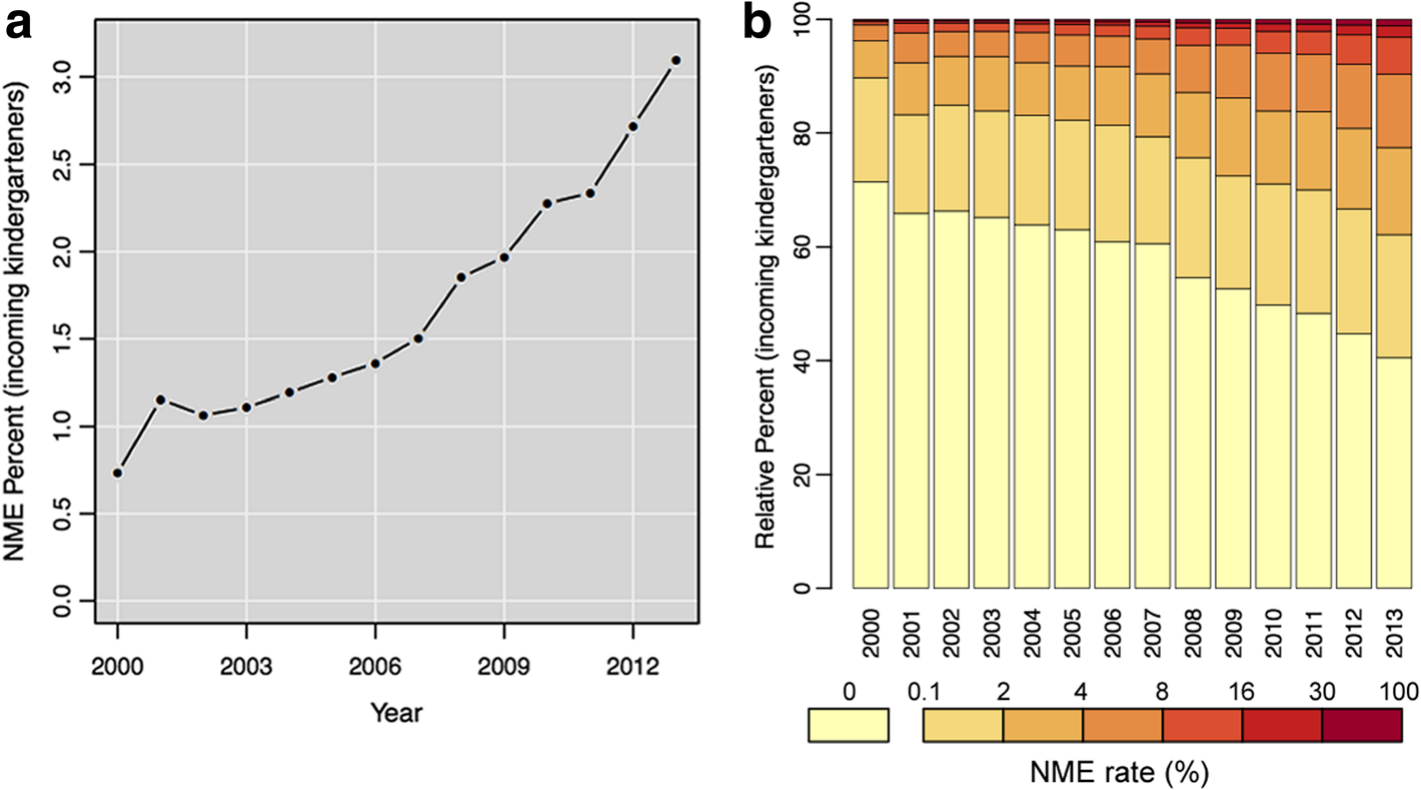
NME rate (%) for kindergarteners in CA for 2000–2013. The overall NME rate (%) is presented in (**a**). The distribution of kindergarteners entering school by the NME rate (%) of the school they attended is presented in (**b**). In (**b**), the relative percent of students is graphed on the x-axis and the NME rate of the school is distinguished using the color of the bar, e.g., over 70% of kindergarteners attended a school with a 0% NME rate in 2000 and roughly 40% of kindergarteners attended a school with a 0% NME rate in 2013

Figure 1a shows the state-level NME rate for 2000–2013 for the schools included in our analysis, which illustrates that NME use increased from 0.73% of all incoming kindergarteners in 2000 to 3.09% in 2013. The relative distribution of California kindergarteners by the NME rate of the school they attended is presented in Fig. 1b, showing how NMEs were distributed within the overall school system. For example, these results show that the percent of children entering a school with a 0% NME rate fell from roughly 70% in 2000 to roughly 40% in 2013. While the percent of children entering a school with a 0.1–2% NME rate was generally stable over the study period (roughly 20%), there were notable increases for all NME rate categories above 4%.

### Global spatial autocorrelation

**Fig. 2:**
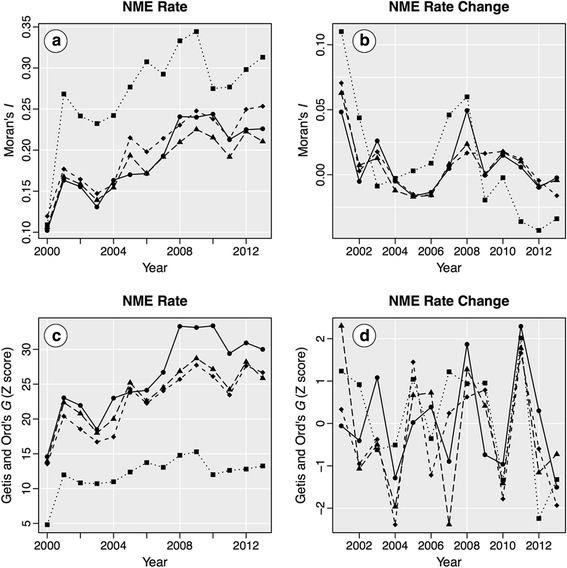
Global spatial autocorrelation results for NME rate and NME rate change for 2000–2013. Data are presented for schools (solid line, circles), BGs (long dashes, triangles), Tracts (medium dashes, diamonds), and SDs (short dashes, squares). Moran’s *I* is presented for (**a**) NME rate and (**b**) NME rate change. Getis and Ord’s *G* is presented for (**c**) NME rate and (**d**) NME rate change

The results of the Moran’s *I* and Getis and Ord’s *G* analysis are presented in Fig. 2. For NME rate, the Moran’s *I* results were positive and showed a steady increase in spatial clustering over the study period for all levels of data aggregation (all tests were statistically significant at *p* < 0.001). Thus, NME rates were clustered in space, and the magnitude of clustering increased over the study period. The Getis and Ord’s *G* results were positive and show a generally increasing trend over the study period. Hence, the spatial clusters were influenced more by regions of high NME values, and that influence increased over the study period. When evaluating *I* and *G* over time, Getis and Ord [32] state that 1) if changes are proportional to the starting values (high starting values increase at a greater rate than low starting values), *I* will increase over time and *G* will be stable and 2) if all observations increase at a similar rate (a constant increase for all regions), *I* will be stable and *G* will increase over time. For NME use from 2000 to 2013, both *I* and *G* increased over time at each level of data aggregation, suggesting that both processes were occurring in California over this time period, e.g., proportional increases with a steady background rise in NME rates as well.

The Moran’s *I* and Getis and Ord’s *G* results for NME rate change for this time period demonstrated very little spatial autocorrelation; thus, while spatial clustering increased over time, the spatial distribution of the year-to-year change in NME use appears to have been largely random. While a few of the *I* and *G* results for NME rate change achieved statistical significance (*p* ≤ 0.05), this result was due to the relatively large number of observations in the data. The overall magnitude of the spatial clustering results was generally quite low, thus the significance of the tests holds no additional interpretive value.

### Local spatial autocorrelation

**Fig. 3:**
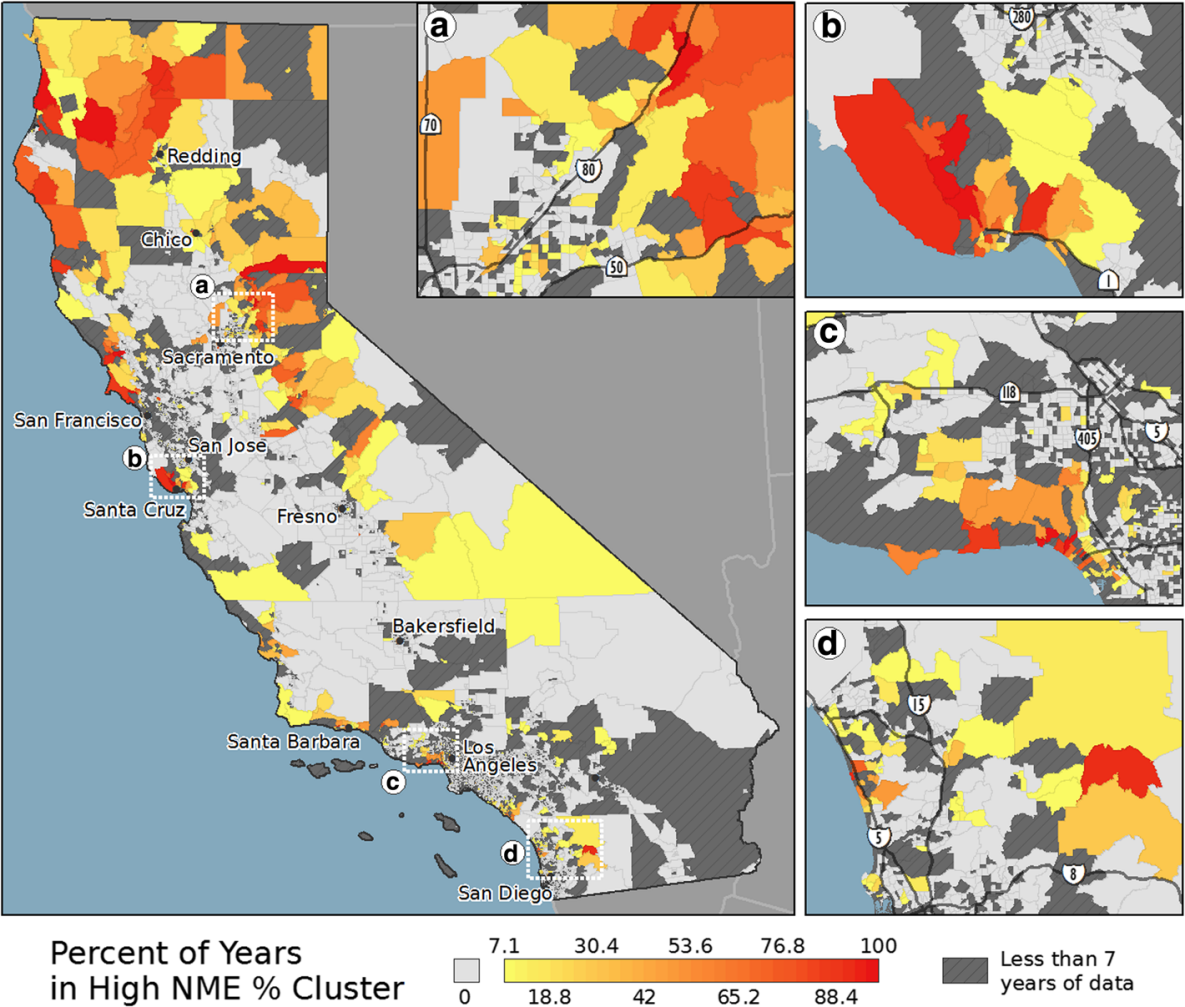
LISA results showing clusters of high NME use for 2000–2013. The mapped values are the percent of years (out of 14) that a region was identified as being in a high NME cluster for 2000–2013. The inset maps show regions near (**a**) Sacramento, (**b**) Santa Cruz and San Jose, (**c**) Los Angeles, and (**d**) San Diego. Only regions having 7 or more years of enrollment data are mapped

The Tract-level results of the LISA analysis can be found in Fig. 3. Tract results are presented, as this level of aggregation allows for mapping and visualization at a state level, but also captures the local variability. The results for the other levels of aggregation (not shown) were similar in nature to the Tract-level results (similar to the results of the global autocorrelation). A notable outcome of this analysis is the relatively small number of regions throughout the state that were identified as being a member of a high NME rate spatial cluster for a large portion of the study period (e.g., > 90%) and their relatively isolated locations across the state. Further visual interpretation of the overall spatial pattern of the High NME clusters suggests that these regions may have acted as seed locations, as the percent of years a region was identified as being in a high NME cluster appears to decrease with increasing distance from these isolated locations (a red, orange, yellow, to grey progression in Fig. 3). Given the statewide increase in NME use over the study period, the most plausible scenario to generate these radial spatial patterns is that the high NME use clusters “grew” in size over time, rather than contracting over time or changing size randomly from year to year. This argument is bolstered by examining the proportion of all Tracts identified as being in a high NME cluster, which steadily increased from 2.76% in 2000 to 4.67% in 2013 (with similar results found for the data aggregated by school, block group, and school district). While this radial spatial pattern can be found throughout California, archetypal examples are present in the northern portion of California (Fig. 3), northeast of Sacramento (Fig. 3a), and west of Los Angeles (Fig. 3c).

**Fig. 4:**
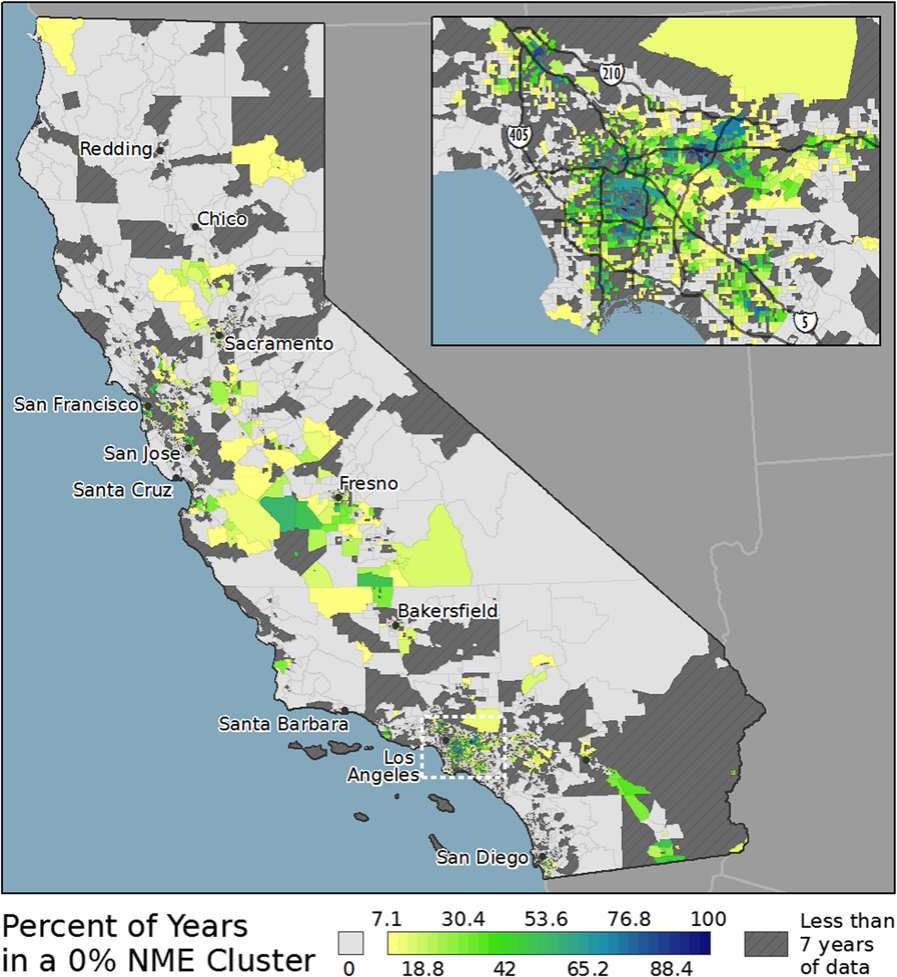
Manual lag results showing clusters of 0% NME use for 2000–2013. The inset map shows a region near Los Angeles. Only regions having 7 or more years of enrollment data are mapped

The manual spatial lag analysis identified regions with a 0% NME rate and a full set of neighbors with a 0% NME rate (0% NME cluster), which are mapped in Fig. 4. The overall distribution of the 0% NME clusters largely resembles a geographic inverse of the High NME use cluster distribution as seen in Fig. 3. Interestingly, these results also demonstrate a similar radial pattern (a blue, green, yellow, to grey progression). Yet, in this case, the most plausible scenario is that the temporal pattern is “reversed,” due to the 0% NME cluster regions shrinking in size throughout the study period. Over the study period, the proportion of all Tracts identified as being in a 0% NME cluster steadily decreased from 20.18% in 2000 to only 3.08% in 2013 (with similar results found for the data aggregated by school, block group, and school district). As NME use increased statewide, it appears to have diffused outward from the initial high use regions into low use regions, thereby causing the 0% NME clusters to contract in extent.

### Grouping analysis

The grouping analysis was carried out separately for each level of data aggregation, which resulted in *k* = 10 groups for the school-level data, *k* = 10 for the block group data, *k* = 9 for the tract data, and *k* = 5 for the school district data. The detailed results of the grouping analysis for all levels of aggregation, including measures of grouping fit and the incremental *F*, can be found in Additional file 1: Table S9-S12. In the main text, only the detailed Tract-level grouping results are presented for consistency with previous results.

**Fig. 5:**
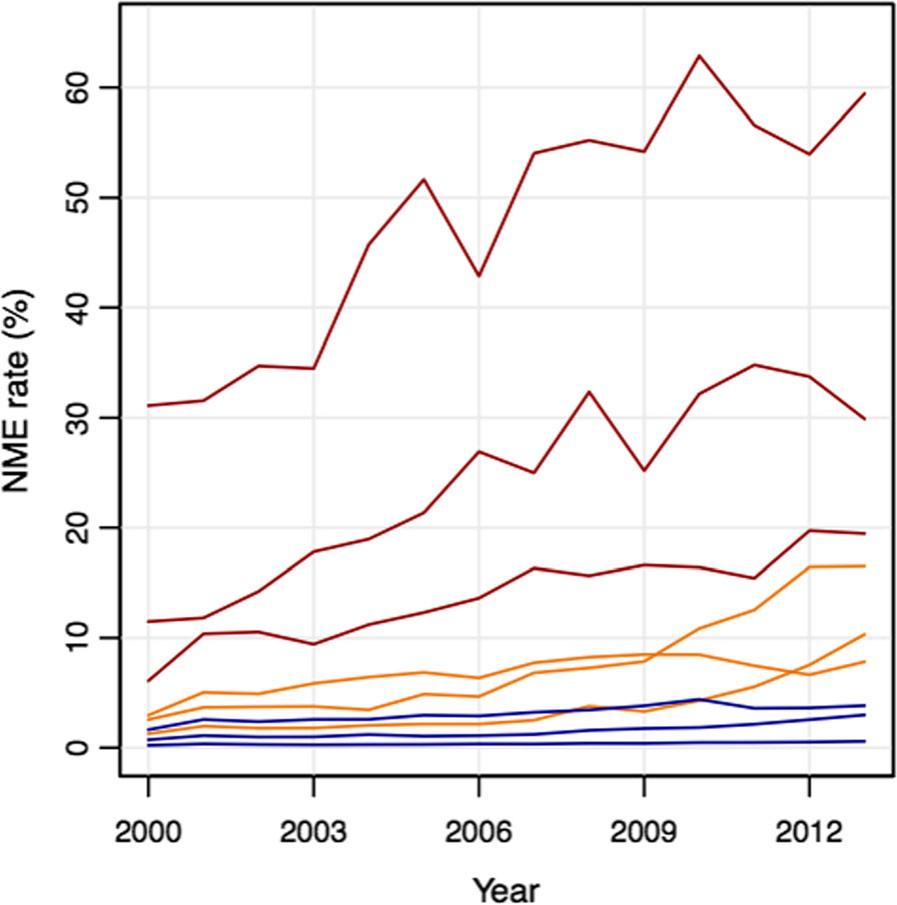
NME rate of the 9 group centers using the Tract-level data. The groups in blue have low NME rates in 2000 with small to moderate increases over the study period. The groups in orange have low NME rates in 2000 and large increases over the study period. The groups in red have high NME rates in 2000 and large increases over the study period

For the Tract-level data, the NME rates over the study period corresponding to the centers of the nine groups are plotted in Fig. 5. The group centers can be considered as the average of the group members and thus represent the overall temporal trend in NME use for each group’s individual members. Table 3 contains additional descriptive information for each of the groups. A notable result of the grouping analysis is that roughly 50% of the tracts were placed into Group 1, which had NME rates hovering near 0% throughout the entire study period. Specifically, during this period of high growth in NME use, roughly half of all regions in California did not demonstrate substantial variation from 0% NME use. Groups 2 and 4 also provide interesting results, as the NME use in these regions was very similar to the state as a whole and represented roughly 35% of all regions. Groups 3, 5, and 6 had generally low NME use in 2000 (< 3%), but large increases throughout the study period. These three groups include roughly 12.4% of all the tracts in California and potentially capture those regions that may have been more influenced by the discussion surrounding vaccines and the changes in nearby vaccine-related behavior than their counterparts in Groups 1, 2, and 4. Finally, Groups 7–9 include those regions having high NME use in 2000 and large increases throughout the study period; these regions were likely the seed locations of vaccine refusal in California (given their high initial NME rates) and also potentially demonstrate regions where vaccine refusal was a self-reinforcing process (given the large increases throughout the study period), such that NME use by some parents in the region appeared to lead more parents in that same region to use NMEs at a later time [17]. However, there are only 72 tracts assigned to these three groups (1.8% of all tracts), which shows that regions having consistently high NME use over this time period were a small proportion of all regions in California as a whole.

**Table 3:** Descriptive information for the 9 groups produced by the grouping analysis

Group	N	N(%)	2000	2013	CHG	Line Color	Description
1	2113	50.6	0.2	0.6	0.4	Blue	Near 0% throughout
2	1017	24.3	0.7	3	2.3	Blue	Starts low, moderate increase (similar to CA)
3	287	6.9	1.2	10.3	9.1	Orange	Starts low, large increase
4	453	10.8	1.6	3.8	2.2	Blue	Starts low, moderate increase (similar to CA)
5	94	2.3	2.5	16.5	14.5	Orange	Starts low, large increase
6	137	3.3	2.9	7.8	4.9	Orange	Starts low, large increase
7	52	1.2	6.1	19.5	13.4	Red	Starts high, large increase
8	20	0.5	11.5	29.9	18.4	Red	Starts high, large increase
9	4	0.1	31.1	59.4	28.3	Red	Starts high, large increase

Groups are based on the Tract-level data. N(%) contains the percent of all tracts in the grouping analysis (*n* = 4177). The fields 2000 and 2013 contain the NME rates (%) of the group centers for those years. CHG contains the absolute change in NME rate (%) from 2000 to 2013. The Line Color field corresponds to the line colors used in Fig. 5 and the Description field contains a short description of the starting NME rate and the change over the study period

**Fig. 6:**
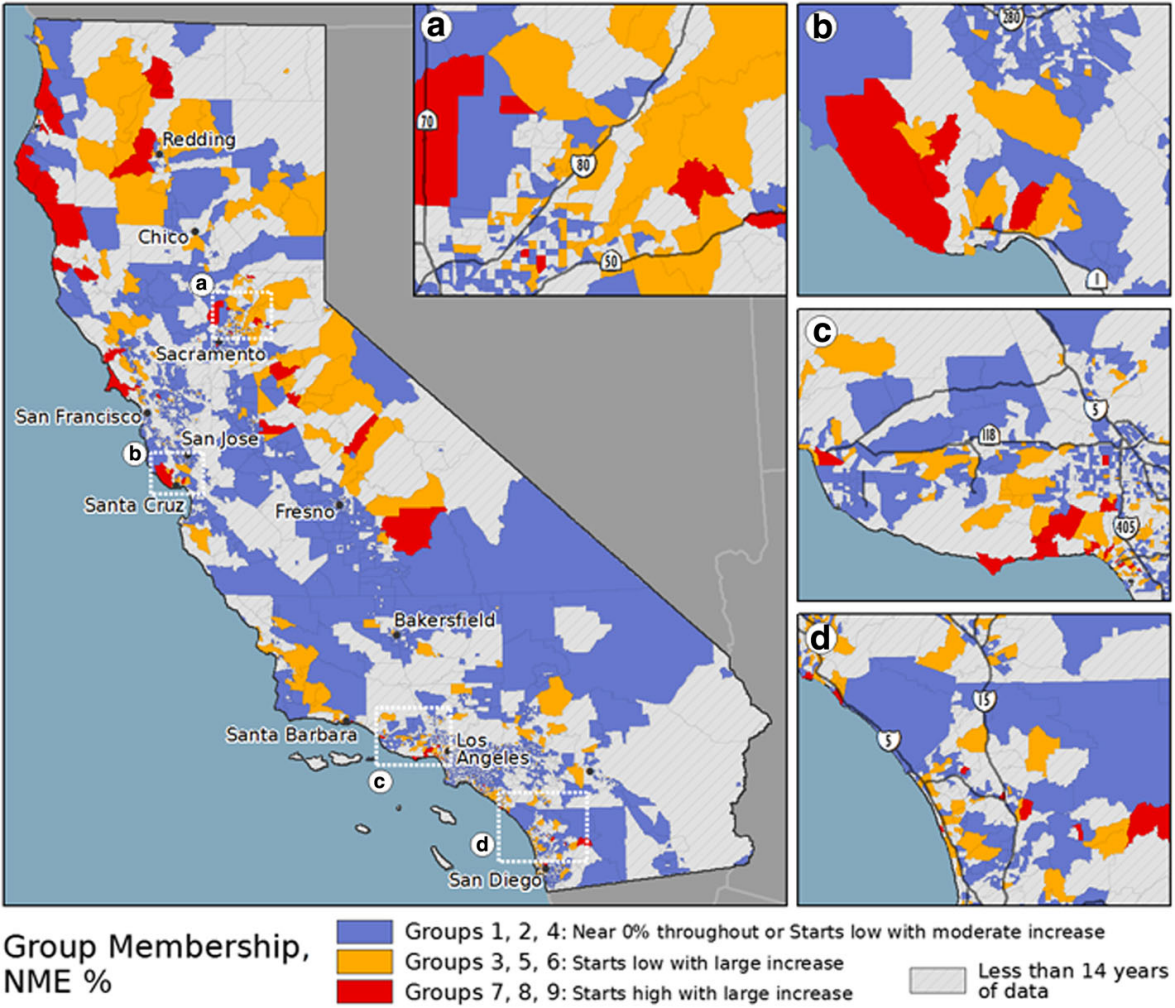
Statewide map of group membership for the Tract-level data. The inset maps show regions near (**a**) Sacramento, (**b**) Santa Cruz and San Jose, (**c**) Los Angeles, and (**d**) San Diego. The groups in blue have low NME rates in 2000 with small to moderate increases over the study period. The groups in orange have low NME rates in 2000 and large increases over the study period. The groups in red have high NME rates in 2000 and large increases over the study period

The group membership of the tracts is mapped in Fig. 6. For visualization purposes, the groups have been classified into the three categories based on the interpretation of the grouping results. Specifically, the categories are seed locations (Groups 7–9), regions with large increases (Groups 3, 5, and 6), and regions with little or moderate increases (Groups 1, 2, and 4). The resulting map reinforces the outcomes from the local clustering analysis. Specifically, in a number of places throughout California (e.g., near Redding, Santa Cruz and San Jose, and Los Angeles), the maps demonstrate a similar radial pattern visible in the High NME cluster maps; the regions having low starting NME rates and large increases (orange) appear to be located near the seed locations (red). This finding suggests, given the interpretation of the various “group” behaviors based on their temporal trends in NME use, that regions with high NME use early in the study period may have influenced their spatial neighbors’ NME use over time.

## Discussion

By evaluating the spatiotemporal patterns of NME use over a 14-year time period for a state-level study area, this analysis helps to shed light on how vaccine refusal evolves over time across a large spatial extent. While NME use in California rose from 0.73% to 3.09% over this time period, our grouping analysis showed that 50% of the regions had consistently low NME rates (at or near 0%) over time. These findings show that the vaccine-related behavior of parents in many California regions was not substantially affected by the changes in NME use occurring in other regions over this time period. Another 35% of all regions had moderate increases in NME use, mirroring the statewide behavior. The remaining 15% of all regions in the state had large increases in NME use over this time period. Hence, the largest increases in vaccine hesitancy appear to have occurred in a relatively small proportion of regions throughout the state. In these regions, parents appeared to be influenced to use NMEs due to the relatively large proportion of other parents that already chose to use NMEs in prior years [17].

Part of understanding the potential risks associated with vaccine refusal is determining where parents choosing NMEs reside, given current understanding how geographic clustering of communities having high NME rates and low vaccination coverage affects herd immunity and VPD outbreak risk. The results of the global clustering analysis clearly demonstrated that NME use in California became more spatially clustered over this time period. The local clustering results provided information regarding how the spatial clusters evolved over time, notably showing the expansion of the High NME use clusters and the contraction of the 0% NME use clusters. Hence, not only did NME use increase statewide over this time period, it became more geographically clustered overall, which produced expanding local clusters of high use. In these local geographic clusters, even if some of the students with NMEs had actually been vaccinated, there was an increased probability that unvaccinated (or undervaccinated) individuals could come into contact with one another, increasing the risk of disease transmission [38] and the potential for outbreaks [39]. This is especially salient when examining school age children, as schools offer an environment where large numbers of children interact on a regular basis. Another notable finding pertaining to disease transmission risk is the contraction of the 0% NME use clusters over time. Particularly, these locations could be viewed as relatively safe haven from potential outbreaks, as there were no students exempted from vaccination requirements at the specific location, nor in its immediate neighborhood. In the year 2000, for example, 27.84% of all schools (*n* = 2017) were identified as being in a 0% NME use cluster, but that number fell to only 5.62% of schools (*n* = 392) by 2013 as NME use expanded geographically.

The local spatial autocorrelation and the grouping analysis also provided interesting results regarding the potential origins of vaccine refusal in California. Notably, the regions with high NME use in the early years of the study period appeared to be small in number and relatively isolated geographically. The observed radial patterns in both the High NME cluster map and the group membership map, suggest that these isolated regions acted as seed locations that stimulated NME use in nearby regions. Specifically, the regions near the seed locations had larger increases in NME use than those further from the seed locations. The corresponding contracting radial pattern in the 0% NME cluster map provided further evidence of the phenomenon. While others have suggested NME use was a spatially diffusive process in California [15, 29], this analysis provided a more rigorous analysis of this hypothesis and produced compelling evidence that spatial diffusion occurred over this time period.

We did not examine whether the observed spatiotemporal patterns were purely due to spatial diffusion or the result of social mechanisms that manifested as spatial patterns. For example, vaccine hesitancy could have expanded within and among groups having similar socioeconomic and demographic characteristics via social, rather than spatial, contacts and the observed spatial patterns are simply a reflection of social sorting or the self-selection of people with similar characteristics or vaccine-related beliefs into proximal geographic regions [17]. By concentrating on geographic proximity, our results do suggest that parents choosing to get an NME may be bolstered by other nearby parents making a similar decision; however, we cannot firmly state whether this process was driven by interactions among parents living in geographically proximal regions (regardless of their social similarity or vaccine-related beliefs), by interactions among parents with similar social characteristics or vaccine-related beliefs who happen to live in geographical proximal regions, or some mixture of both. Future research that integrates socioeconomic or demographic similarity or social connections among regions may help to disentangle the roles that local spatial processes and social processes played in the overall increase in NME use and the observed spatiotemporal patterns.

Our analysis was conducted at multiple levels of data aggregation in an effort to assess the potential effects of the Modifiable Areal Unit Problem, wherein the results of statistical tests are sensitive to the scale of the observation units when using aggregated spatial data [40]. While we focused on the Tract-level results, the results of the School, Block Group, and School District analyses were remarkably similar. This outcome supports our overall findings, as it demonstrates that the results were consistent across the multiple scales of analysis. Yet, the consistency also limits our ability to evaluate whether there is a spatial scale (e.g., family, school, local neighborhood, school district) at which the diffusion of vaccine refusal may operate; however, it does provide an interesting opportunity for future research.

One matter that was not considered in our analysis is the potential effect of school practices on the observed spatiotemporal variations in NME rates over time. Compliance enforcement and practices have been shown to be associated with school vaccinations rates [41]. Prior to the implementation of AB2109 in 2014, NMEs were relatively easy to claim, and some California schools (11% of the 298 schools surveyed) offered a NME as a “convenience” option for parents of children not fully up to date on the school-entry vaccinations or that could not provide their child’s required vaccination records [42]. While the vaccine-related beliefs of the parents were not assessed in the survey, an assumption is that these NMEs were potentially driven by the 1) the burden on parents of producing the required documentation or 2) the administrative burden on schools of offering a conditional admittance, which requires the school to follow-up at a later date [29]. Increases in this school-level practice could account for some of the observed spatiotemporal patterns of NME use, especially if it were co-located in regions where vaccine hesitancy in parents was increasing. The effect of schools on NME use presents another interesting opportunity for future research.

Due to the cross-sectional nature of the data, our analysis was not able to evaluate whether parents “progressed” along the spectrum of vaccine hesitancy during the time period. However, we were able to evaluate how vaccine refusal evolved as a geographic phenomenon over this time period. We found that early in the study period, the number of regions having a high proportion of vaccine refusers was relatively small in number and these regions were isolated geographically throughout the state. Further, the use of NMEs in these isolated regions appeared to stimulate vaccine refusal in nearby regions, as the corresponding maps of NME use demonstrated a radial spatial pattern throughout much of the state.

## Conclusions

This analysis aimed to understand the spatiotemporal evolution of vaccine refusal. We examined NMEs over a 14-year time period in California that was devoid of changes to large-scale vaccine-related policy in an effort to focus on how the changes in vaccine-related behavior manifested in space over time. Our results showed that there was an observable spatial structure to vaccine refusal and NME use in California over this time period. Notably, vaccine refusal appeared to be 1) a self-reinforcing process, such that communities with the highest NME use also had the largest increases in NME use throughout the study period and 2) a spatially diffusive process, such that regions located nearby those with high initial NME use demonstrated larger increases in NME use over time than regions located further from the initial high use regions. While our specific findings are restricted to California, they do potentially provide important information for other states and countries that are struggling with increases in vaccine refusal and declining childhood vaccination coverage. Specifically, our results demonstrate that efforts aimed at decreasing future NME use may be most effective if they are not only focused on regions where NME use is already high, but also on those located near to the high use regions, as they may be susceptible to future increases in vaccine refusal.

## Additional file

Additional file 1: Additional results from the neighborhood definition and grouping analysis. (DOCX 78 kb)

## Abbreviations

BG: Block group
CDPH: California Department of Public Health
ID: Inverse distance
KNN: K nearest neighbors
LISA: Local Indicator of Spatial Association
NME: Nonmedical exemption
SD: School district
US: United States
VPD: Vaccine-preventable disease

## Footnotes

1 The District of Colombia is referred to as a state for the remainder of this document.

2 For the remainder to the manuscript, specific school years will be referred to using the year in which students entered kindergarten, e.g., 2013–2014 will simply be referred to as 2013.
