# Supplementary material for: Examining the spatiotemporal evolution of vaccine refusal: nonmedical exemptions from vaccination in California, 2000–2013
Source: BMC Public Health. 2018 Apr 24;18:458. doi: 10.1186/s12889-018-5368-y (PMC5913803; doi:10.1186/s12889-018-5368-y)
Supplement: Supplementary file 1 — Additional results from the neighborhood definition and grouping analysis. (DOCX 78 kb) [file 12889_2018_5368_MOESM1_ESM.docx]

**Examining the spatiotemporal evolution of vaccine refusal: Nonmedical exemptions from vaccination in California, 2000-2013**

**Additional File 1**

Paul L. Delamater, Timothy F. Leslie, and Y. Tony Yang

**Table S1. Moran’s *I* values for NME rate (%) for school-level observations, under eight neighborhood definitions.**

| **YEAR** | **ID(5)** | **ID(10)** | **ID(15)** | **ID(20)** | **KNN(5)** | **KNN(10)** | **KNN(15)** | **KNN(20)** |
| --- | --- | --- | --- | --- | --- | --- | --- | --- |
| 2000 | 0.080 | 0.083 | 0.083 | 0.078 | 0.102 | 0.092 | 0.081 | 0.073 |
| 2001 | 0.145 | 0.155 | 0.155 | 0.152 | 0.163 | 0.147 | 0.130 | 0.121 |
| 2002 | 0.148 | 0.152 | 0.152 | 0.146 | 0.155 | 0.134 | 0.120 | 0.115 |
| 2003 | 0.098 | 0.110 | 0.100 | 0.097 | 0.131 | 0.105 | 0.099 | 0.093 |
| 2004 | 0.129 | 0.142 | 0.148 | 0.135 | 0.163 | 0.133 | 0.126 | 0.122 |
| 2005 | 0.122 | 0.139 | 0.139 | 0.127 | 0.170 | 0.160 | 0.150 | 0.138 |
| 2006 | 0.159 | 0.167 | 0.160 | 0.150 | 0.171 | 0.155 | 0.141 | 0.132 |
| 2007 | 0.156 | 0.172 | 0.165 | 0.152 | 0.193 | 0.175 | 0.157 | 0.144 |
| 2008 | 0.203 | 0.209 | 0.201 | 0.194 | 0.241 | 0.209 | 0.182 | 0.167 |
| 2009 | 0.213 | 0.218 | 0.203 | 0.192 | 0.240 | 0.196 | 0.171 | 0.155 |
| 2010 | 0.214 | 0.226 | 0.211 | 0.197 | 0.244 | 0.212 | 0.185 | 0.173 |
| 2011 | 0.179 | 0.184 | 0.180 | 0.169 | 0.213 | 0.197 | 0.176 | 0.167 |
| 2012 | 0.179 | 0.180 | 0.176 | 0.168 | 0.225 | 0.202 | 0.182 | 0.174 |
| 2013 | 0.203 | 0.208 | 0.202 | 0.195 | 0.226 | 0.206 | 0.185 | 0.175 |

**Table S2. Moran’s *I* values for NME rate (%) for block group observations, under ten neighborhood definitions.**

| **YEAR** | **ID(5)** | **ID(10)** | **ID(15)** | **ID(20)** | **KNN(5)** | **KNN(10)** | **KNN(15)** | **KNN(20)** | **CON(Q)** | **CON(R)** |
| --- | --- | --- | --- | --- | --- | --- | --- | --- | --- | --- |
| 2000 | 0.073 | 0.076 | 0.078 | 0.079 | 0.106 | 0.093 | 0.083 | 0.079 | 0.119 | 0.104 |
| 2001 | 0.105 | 0.125 | 0.126 | 0.116 | 0.167 | 0.155 | 0.138 | 0.129 | 0.128 | 0.119 |
| 2002 | 0.104 | 0.110 | 0.119 | 0.116 | 0.159 | 0.143 | 0.127 | 0.119 | 0.137 | 0.122 |
| 2003 | 0.089 | 0.101 | 0.103 | 0.102 | 0.139 | 0.125 | 0.114 | 0.106 | 0.130 | 0.116 |
| 2004 | 0.093 | 0.117 | 0.111 | 0.102 | 0.154 | 0.138 | 0.127 | 0.119 | 0.120 | 0.117 |
| 2005 | 0.119 | 0.136 | 0.137 | 0.128 | 0.193 | 0.165 | 0.157 | 0.148 | 0.150 | 0.131 |
| 2006 | 0.125 | 0.129 | 0.131 | 0.121 | 0.172 | 0.152 | 0.135 | 0.133 | 0.140 | 0.109 |
| 2007 | 0.138 | 0.146 | 0.150 | 0.142 | 0.192 | 0.173 | 0.156 | 0.147 | 0.139 | 0.117 |
| 2008 | 0.134 | 0.170 | 0.170 | 0.161 | 0.210 | 0.193 | 0.176 | 0.169 | 0.164 | 0.144 |
| 2009 | 0.179 | 0.191 | 0.170 | 0.156 | 0.225 | 0.192 | 0.174 | 0.163 | 0.192 | 0.166 |
| 2010 | 0.150 | 0.173 | 0.164 | 0.154 | 0.215 | 0.189 | 0.172 | 0.164 | 0.178 | 0.151 |
| 2011 | 0.142 | 0.158 | 0.157 | 0.148 | 0.192 | 0.184 | 0.166 | 0.159 | 0.149 | 0.135 |
| 2012 | 0.171 | 0.176 | 0.166 | 0.160 | 0.223 | 0.201 | 0.182 | 0.174 | 0.191 | 0.161 |
| 2013 | 0.148 | 0.164 | 0.155 | 0.150 | 0.211 | 0.193 | 0.183 | 0.176 | 0.169 | 0.145 |

**Table S3. Moran’s *I* values for NME rate (%) for tract observations, under ten neighborhood definitions.**

| **YEAR** | **ID(5)** | **ID(10)** | **ID(15)** | **ID(20)** | **KNN(5)** | **KNN(10)** | **KNN(15)** | **KNN(20)** | **CON(Q)** | **CON(R)** |
| --- | --- | --- | --- | --- | --- | --- | --- | --- | --- | --- |
| 2000 | 0.078 | 0.093 | 0.093 | 0.087 | 0.120 | 0.110 | 0.097 | 0.091 | 0.133 | 0.136 |
| 2001 | 0.108 | 0.121 | 0.120 | 0.126 | 0.177 | 0.157 | 0.146 | 0.141 | 0.196 | 0.195 |
| 2002 | 0.103 | 0.117 | 0.122 | 0.118 | 0.164 | 0.152 | 0.139 | 0.134 | 0.171 | 0.173 |
| 2003 | 0.087 | 0.097 | 0.101 | 0.101 | 0.147 | 0.120 | 0.111 | 0.101 | 0.158 | 0.162 |
| 2004 | 0.088 | 0.106 | 0.110 | 0.110 | 0.158 | 0.142 | 0.132 | 0.121 | 0.170 | 0.173 |
| 2005 | 0.145 | 0.146 | 0.150 | 0.143 | 0.215 | 0.190 | 0.178 | 0.163 | 0.232 | 0.231 |
| 2006 | 0.157 | 0.142 | 0.157 | 0.148 | 0.198 | 0.178 | 0.160 | 0.155 | 0.228 | 0.223 |
| 2007 | 0.172 | 0.160 | 0.160 | 0.160 | 0.214 | 0.197 | 0.182 | 0.172 | 0.229 | 0.226 |
| 2008 | 0.182 | 0.159 | 0.165 | 0.170 | 0.231 | 0.200 | 0.188 | 0.181 | 0.259 | 0.259 |
| 2009 | 0.220 | 0.193 | 0.182 | 0.189 | 0.248 | 0.213 | 0.192 | 0.177 | 0.271 | 0.266 |
| 2010 | 0.181 | 0.178 | 0.178 | 0.166 | 0.238 | 0.210 | 0.190 | 0.180 | 0.266 | 0.267 |
| 2011 | 0.165 | 0.156 | 0.154 | 0.153 | 0.214 | 0.191 | 0.175 | 0.167 | 0.215 | 0.211 |
| 2012 | 0.190 | 0.204 | 0.187 | 0.189 | 0.250 | 0.224 | 0.206 | 0.196 | 0.250 | 0.253 |
| 2013 | 0.183 | 0.191 | 0.183 | 0.182 | 0.253 | 0.229 | 0.213 | 0.204 | 0.270 | 0.266 |

**Table S4. Moran’s *I* values for NME rate (%) for school district observations, under ten neighborhood definitions.**

| **YEAR** | **ID(5)** | **ID(10)** | **ID(15)** | **ID(20)** | **KNN(5)** | **KNN(10)** | **KNN(15)** | **KNN(20)** | **CON(Q)** | **CON(R)** |
| --- | --- | --- | --- | --- | --- | --- | --- | --- | --- | --- |
| 2000 | 0.045 | 0.061 | 0.098 | 0.107 | 0.109 | 0.115 | 0.087 | 0.085 | 0.137 | 0.136 |
| 2001 | 0.056 | 0.110 | 0.149 | 0.167 | 0.268 | 0.219 | 0.177 | 0.170 | 0.296 | 0.295 |
| 2002 | 0.042 | 0.113 | 0.187 | 0.183 | 0.242 | 0.203 | 0.167 | 0.141 | 0.225 | 0.224 |
| 2003 | 0.040 | 0.164 | 0.185 | 0.218 | 0.232 | 0.185 | 0.154 | 0.141 | 0.210 | 0.209 |
| 2004 | 0.035 | 0.142 | 0.270 | 0.250 | 0.242 | 0.182 | 0.157 | 0.142 | 0.223 | 0.222 |
| 2005 | 0.066 | 0.241 | 0.255 | 0.254 | 0.277 | 0.238 | 0.211 | 0.183 | 0.268 | 0.267 |
| 2006 | 0.090 | 0.167 | 0.262 | 0.269 | 0.308 | 0.225 | 0.195 | 0.183 | 0.298 | 0.298 |
| 2007 | 0.079 | 0.162 | 0.217 | 0.237 | 0.293 | 0.236 | 0.194 | 0.176 | 0.293 | 0.294 |
| 2008 | 0.061 | 0.173 | 0.275 | 0.298 | 0.333 | 0.264 | 0.226 | 0.196 | 0.319 | 0.321 |
| 2009 | 0.128 | 0.210 | 0.311 | 0.311 | 0.344 | 0.295 | 0.238 | 0.212 | 0.373 | 0.372 |
| 2010 | 0.066 | 0.206 | 0.260 | 0.265 | 0.275 | 0.208 | 0.194 | 0.177 | 0.297 | 0.297 |
| 2011 | 0.067 | 0.171 | 0.236 | 0.242 | 0.277 | 0.222 | 0.181 | 0.169 | 0.258 | 0.261 |
| 2012 | 0.067 | 0.195 | 0.222 | 0.255 | 0.298 | 0.275 | 0.240 | 0.217 | 0.297 | 0.298 |
| 2013 | 0.066 | 0.178 | 0.253 | 0.288 | 0.313 | 0.261 | 0.222 | 0.208 | 0.323 | 0.323 |

**Table S5. Pearson’s *R* correlation values for Moran’s *I* values (2000-2013) for school-level observations.** The “Mean” row contains the average correlation among the neighborhood definitions.

|  | **ID(5)** | **ID(10)** | **ID(15)** | **ID(20)** | **KNN(5)** | **KNN(10)** | **KNN(15)** | **KNN(20)** |
| --- | --- | --- | --- | --- | --- | --- | --- | --- |
| ID(5) |  | 0.994 | 0.986 | 0.989 | 0.964 | 0.939 | 0.915 | 0.905 |
| ID(10) | 0.994 |  | 0.992 | 0.988 | 0.969 | 0.944 | 0.925 | 0.912 |
| ID(15) | 0.986 | 0.992 |  | 0.995 | 0.962 | 0.946 | 0.932 | 0.925 |
| ID(20) | 0.989 | 0.988 | 0.995 |  | 0.954 | 0.938 | 0.920 | 0.914 |
| KNN(5) | 0.964 | 0.969 | 0.962 | 0.954 |  | 0.981 | 0.970 | 0.959 |
| KNN(10) | 0.939 | 0.944 | 0.946 | 0.938 | 0.981 |  | 0.995 | 0.986 |
| KNN(15) | 0.915 | 0.925 | 0.932 | 0.920 | 0.970 | 0.995 |  | 0.996 |
| KNN(20) | 0.905 | 0.912 | 0.925 | 0.914 | 0.959 | 0.986 | 0.996 |  |
| *Mean* | *0.956* | *0.961* | *0.963* | *0.957* | *0.966* | *0.961* | *0.951* | *0.942* |

**Table S6. Pearson’s *R* correlation values for Moran’s *I* values (2000-2013) for block group observations.** The “Mean” row contains the average correlation among the neighborhood definitions.

|  | **ID(5)** | **ID(10)** | **ID(15)** | **ID(20)** | **KNN(5)** | **KNN(10)** | **KNN(15)** | **KNN(20)** | **CON(Q)** | **CON(R)** |
| --- | --- | --- | --- | --- | --- | --- | --- | --- | --- | --- |
| **ID(5)** |  | 0.960 | 0.936 | 0.932 | 0.945 | 0.926 | 0.908 | 0.905 | 0.926 | 0.874 |
| **ID(10)** | 0.960 |  | 0.980 | 0.969 | 0.979 | 0.972 | 0.962 | 0.956 | 0.904 | 0.900 |
| **ID(15)** | 0.936 | 0.980 |  | 0.993 | 0.980 | 0.987 | 0.972 | 0.970 | 0.858 | 0.841 |
| **ID(20)** | 0.932 | 0.969 | 0.993 |  | 0.971 | 0.985 | 0.972 | 0.972 | 0.873 | 0.850 |
| **KNN(5)** | 0.945 | 0.979 | 0.980 | 0.971 |  | 0.984 | 0.983 | 0.978 | 0.894 | 0.876 |
| **KNN(10)** | 0.926 | 0.972 | 0.987 | 0.985 | 0.984 |  | 0.994 | 0.992 | 0.851 | 0.846 |
| **KNN(15)** | 0.908 | 0.962 | 0.972 | 0.972 | 0.983 | 0.994 |  | 0.998 | 0.848 | 0.849 |
| **KNN(20)** | 0.905 | 0.956 | 0.970 | 0.972 | 0.978 | 0.992 | 0.998 |  | 0.842 | 0.831 |
| **CON(Q)** | 0.926 | 0.904 | 0.858 | 0.873 | 0.894 | 0.851 | 0.848 | 0.842 |  | 0.961 |
| **CON(R)** | 0.874 | 0.900 | 0.841 | 0.850 | 0.876 | 0.846 | 0.849 | 0.831 | 0.961 |  |
| *Mean* | *0.924* | *0.954* | *0.946* | *0.946* | *0.954* | *0.949* | *0.943* | *0.938* | *0.884* | *0.870* |

**Table S7. Pearson’s *R* correlation values for Moran’s *I* values (2000-2013) for tract observations.** The “Mean” row contains the average correlation among the neighborhood definitions.

|  | **ID(5)** | **ID(10)** | **ID(15)** | **ID(20)** | **KNN(5)** | **KNN(10)** | **KNN(15)** | **KNN(20)** | **CON(Q)** | **CON(R)** |
| --- | --- | --- | --- | --- | --- | --- | --- | --- | --- | --- |
| **ID(5)** |  | 0.956 | 0.973 | 0.976 | 0.957 | 0.944 | 0.928 | 0.920 | 0.953 | 0.946 |
| **ID(10)** | 0.956 |  | 0.981 | 0.982 | 0.973 | 0.975 | 0.961 | 0.954 | 0.935 | 0.935 |
| **ID(15)** | 0.973 | 0.981 |  | 0.986 | 0.983 | 0.985 | 0.973 | 0.969 | 0.969 | 0.967 |
| **ID(20)** | 0.976 | 0.982 | 0.986 |  | 0.985 | 0.980 | 0.972 | 0.968 | 0.964 | 0.961 |
| **KNN(5)** | 0.957 | 0.973 | 0.983 | 0.985 |  | 0.991 | 0.989 | 0.980 | 0.980 | 0.979 |
| **KNN(10)** | 0.944 | 0.975 | 0.985 | 0.980 | 0.991 |  | 0.997 | 0.993 | 0.964 | 0.962 |
| **KNN(15)** | 0.928 | 0.961 | 0.973 | 0.972 | 0.989 | 0.997 |  | 0.996 | 0.960 | 0.959 |
| **KNN(20)** | 0.920 | 0.954 | 0.969 | 0.968 | 0.980 | 0.993 | 0.996 |  | 0.953 | 0.951 |
| **CON(Q)** | 0.953 | 0.935 | 0.969 | 0.964 | 0.980 | 0.964 | 0.960 | 0.953 |  | 0.998 |
| **CON(R)** | 0.946 | 0.935 | 0.967 | 0.961 | 0.979 | 0.962 | 0.959 | 0.951 | 0.998 |  |
| *Mean* | *0.950* | *0.961* | *0.976* | *0.975* | *0.980* | *0.977* | *0.970* | *0.965* | *0.964* | *0.962* |

**Table S8. Pearson’s *R* correlation values for Moran’s *I* values (2000-2013) for school district observations.** The “Mean” row contains the average correlation among the neighborhood definitions.

|  | **ID(5)** | **ID(10)** | **ID(15)** | **ID(20)** | **KNN(5)** | **KNN(10)** | **KNN(15)** | **KNN(20)** | **CON(Q)** | **CON(R)** |
| --- | --- | --- | --- | --- | --- | --- | --- | --- | --- | --- |
| **ID(5)** |  | 0.524 | 0.565 | 0.581 | 0.643 | 0.681 | 0.620 | 0.642 | 0.757 | 0.756 |
| **ID(10)** | 0.524 |  | 0.794 | 0.833 | 0.724 | 0.717 | 0.800 | 0.767 | 0.660 | 0.662 |
| **ID(15)** | 0.565 | 0.794 |  | 0.958 | 0.810 | 0.705 | 0.755 | 0.720 | 0.724 | 0.725 |
| **ID(20)** | 0.581 | 0.833 | 0.958 |  | 0.884 | 0.806 | 0.852 | 0.835 | 0.803 | 0.806 |
| **KNN(5)** | 0.643 | 0.724 | 0.810 | 0.884 |  | 0.940 | 0.943 | 0.938 | 0.946 | 0.948 |
| **KNN(10)** | 0.681 | 0.717 | 0.705 | 0.806 | 0.940 |  | 0.979 | 0.968 | 0.925 | 0.929 |
| **KNN(15)** | 0.620 | 0.800 | 0.755 | 0.852 | 0.943 | 0.979 |  | 0.986 | 0.915 | 0.919 |
| **KNN(20)** | 0.642 | 0.767 | 0.720 | 0.835 | 0.938 | 0.968 | 0.986 |  | 0.933 | 0.937 |
| **CON(Q)** | 0.757 | 0.660 | 0.724 | 0.803 | 0.946 | 0.925 | 0.915 | 0.933 |  | 1.000 |
| **CON(R)** | 0.756 | 0.662 | 0.725 | 0.806 | 0.948 | 0.929 | 0.919 | 0.937 | 1.000 |  |
| *Mean* | *0.641* | *0.720* | *0.751* | *0.818* | *0.864* | *0.850* | *0.863* | *0.858* | *0.851* | *0.853* |

**Table S9. Measures of grouping fit for group solutions *k* = 2-30 for School observations.** WSS is within group sum of squared errors, the amount of variation not captured by the groups. BSS is the between group sum of squared errors, the amount of variation captured by the groups. R^2^ is the coefficient of determination, which is calculated by dividing the BSS value by the total sum of squared errors (WSS + BSS). The incremental *F* score (inc *F*) captures the amount of explanatory power gained by adding another group to the solution (while penalizing for adding that group).

| **Groups (*k*)** | **WSS** | **BSS** | **R^2^** | **inc *F*** |
| --- | --- | --- | --- | --- |
| 2 | 87.40 | 59.17 | 0.40 | NA |
| 3 | 66.11 | 80.47 | 0.55 | 1609.94 |
| 4 | 55.25 | 91.32 | 0.62 | 981.46 |
| 5 | 49.86 | 96.71 | 0.66 | 540.16 |
| 6 | 47.60 | 98.98 | 0.68 | 237.73 |
| 7 | 46.19 | 100.39 | 0.68 | 152.37 |
| 8 | 44.86 | 101.72 | 0.69 | 148.03 |
| 9 | 43.97 | 102.60 | 0.70 | 100.59 |
| 10 | 42.62 | 103.96 | 0.71 | 158.87 |
| 11 | 41.83 | 104.74 | 0.71 | 93.62 |
| 12 | 41.34 | 105.24 | 0.72 | 59.35 |
| 13 | 40.64 | 105.93 | 0.72 | 85.39 |
| 14 | 40.07 | 106.50 | 0.73 | 71.07 |
| 15 | 39.61 | 106.97 | 0.73 | 58.33 |
| 16 | 38.89 | 107.69 | 0.73 | 92.11 |
| 17 | 38.58 | 108.00 | 0.74 | 40.03 |
| 18 | 38.22 | 108.35 | 0.74 | 46.68 |
| 19 | 37.77 | 108.81 | 0.74 | 59.66 |
| 20 | 37.35 | 109.22 | 0.75 | 55.52 |
| 21 | 36.69 | 109.88 | 0.75 | 89.49 |
| 22 | 36.30 | 110.28 | 0.75 | 54.57 |
| 23 | 36.24 | 110.33 | 0.75 | 7.36 |
| 24 | 35.96 | 110.62 | 0.75 | 39.19 |
| 25 | 34.79 | 111.78 | 0.76 | 167.17 |
| 26 | 34.31 | 112.27 | 0.77 | 70.44 |
| 27 | 34.19 | 112.38 | 0.77 | 16.56 |
| 28 | 33.68 | 112.90 | 0.77 | 75.98 |
| 29 | 33.72 | 112.86 | 0.77 | -5.53 |
| 30 | 33.45 | 113.12 | 0.77 | 39.17 |

**Table S10. Measures of grouping fit for group solutions *k* = 2-30 for Block Group observations.** WSS is within group sum of squared errors, the amount of variation not captured by the groups. BSS is the between group sum of squared errors, the amount of variation captured by the groups. R^2^ is the coefficient of determination, which is calculated by dividing the BSS value by the total sum of squared errors (WSS + BSS). The incremental *F* score (inc *F*) captures the amount of explanatory power gained by adding another group to the solution (while penalizing for adding that group).

| **Groups (*k*)** | **WSS** | **BSS** | **R^2^** | **inc *F*** |
| --- | --- | --- | --- | --- |
| 2 | 86.04 | 54.61 | 0.39 | NA |
| 3 | 63.92 | 76.73 | 0.55 | 1692.18 |
| 4 | 54.62 | 86.03 | 0.61 | 832.68 |
| 5 | 49.15 | 91.51 | 0.65 | 544.08 |
| 6 | 46.63 | 94.03 | 0.67 | 264.43 |
| 7 | 45.26 | 95.39 | 0.68 | 147.29 |
| 8 | 43.96 | 96.70 | 0.69 | 144.92 |
| 9 | 42.80 | 97.85 | 0.70 | 131.48 |
| 10 | 41.63 | 99.02 | 0.70 | 137.23 |
| 11 | 41.05 | 99.60 | 0.71 | 69.49 |
| 12 | 40.46 | 100.19 | 0.71 | 70.75 |
| 13 | 39.72 | 100.94 | 0.72 | 91.73 |
| 14 | 39.05 | 101.60 | 0.72 | 82.96 |
| 15 | 38.58 | 102.07 | 0.73 | 59.30 |
| 16 | 38.02 | 102.63 | 0.73 | 71.82 |
| 17 | 37.66 | 102.99 | 0.73 | 46.88 |
| 18 | 37.45 | 103.20 | 0.73 | 27.49 |
| 19 | 37.05 | 103.60 | 0.74 | 52.12 |
| 20 | 36.54 | 104.11 | 0.74 | 68.06 |
| 21 | 36.03 | 104.63 | 0.74 | 69.92 |
| 22 | 35.91 | 104.74 | 0.74 | 15.46 |
| 23 | 35.61 | 105.05 | 0.75 | 41.84 |
| 24 | 35.35 | 105.31 | 0.75 | 35.74 |
| 25 | 35.14 | 105.51 | 0.75 | 28.15 |
| 26 | 34.67 | 105.98 | 0.75 | 66.57 |
| 27 | 34.48 | 106.18 | 0.75 | 27.39 |
| 28 | 34.29 | 106.37 | 0.76 | 26.77 |
| 29 | 33.43 | 107.22 | 0.76 | 124.48 |
| 30 | 33.35 | 107.31 | 0.76 | 12.39 |

**Table S11. Measures of grouping fit for group solutions *k* = 2-30 for Tract observations.** WSS is within group sum of squared errors, the amount of variation not captured by the groups. BSS is the between group sum of squared errors, the amount of variation captured by the groups. R^2^ is the coefficient of determination, which is calculated by dividing the BSS value by the total sum of squared errors (WSS + BSS). The incremental *F* score (inc *F*) captures the amount of explanatory power gained by adding another group to the solution (while penalizing for adding that group).

| **Groups (*k*)** | **WSS** | **BSS** | **R^2^** | **inc *F*** |
| --- | --- | --- | --- | --- |
| 2 | 56.77 | 33.21 | 0.37 | NA |
| 3 | 43.32 | 46.66 | 0.52 | 1295.92 |
| 4 | 37.30 | 52.68 | 0.59 | 673.84 |
| 5 | 34.08 | 55.90 | 0.62 | 394.56 |
| 6 | 32.46 | 57.52 | 0.64 | 208.09 |
| 7 | 31.46 | 58.52 | 0.65 | 132.44 |
| 8 | 30.72 | 59.26 | 0.66 | 99.98 |
| 9 | 29.01 | 60.97 | 0.68 | 246.01 |
| 10 | 28.49 | 61.50 | 0.68 | 76.70 |
| 11 | 27.52 | 62.47 | 0.69 | 146.87 |
| 12 | 27.07 | 62.92 | 0.70 | 69.13 |
| 13 | 26.54 | 63.44 | 0.71 | 82.68 |
| 14 | 26.10 | 63.89 | 0.71 | 71.21 |
| 15 | 25.80 | 64.19 | 0.71 | 48.03 |
| 16 | 25.44 | 64.55 | 0.72 | 59.04 |
| 17 | 25.06 | 64.93 | 0.72 | 62.84 |
| 18 | 24.80 | 65.19 | 0.72 | 44.09 |
| 19 | 24.68 | 65.30 | 0.73 | 19.15 |
| 20 | 24.33 | 65.65 | 0.73 | 59.65 |
| 21 | 24.03 | 65.96 | 0.73 | 53.19 |
| 22 | 23.78 | 66.21 | 0.74 | 43.78 |
| 23 | 23.52 | 66.47 | 0.74 | 45.79 |
| 24 | 23.34 | 66.64 | 0.74 | 31.54 |
| 25 | 23.21 | 66.77 | 0.74 | 22.52 |
| 26 | 23.00 | 66.99 | 0.74 | 39.06 |
| 27 | 22.71 | 67.27 | 0.75 | 51.87 |
| 28 | 22.24 | 67.74 | 0.75 | 87.46 |
| 29 | 22.14 | 67.85 | 0.75 | 20.45 |
| 30 | 22.06 | 67.93 | 0.75 | 14.44 |

**Table S12. Measures of grouping fit for group solutions *k* = 2-30 for School District observations.** WSS is within group sum of squared errors, the amount of variation not captured by the groups. BSS is the between group sum of squared errors, the amount of variation captured by the groups. R^2^ is the coefficient of determination, which is calculated by dividing the BSS value by the total sum of squared errors (WSS + BSS). The incremental *F* score (inc *F*) captures the amount of explanatory power gained by adding another group to the solution (while penalizing for adding that group).

| **Groups (*k*)** | **WSS** | **BSS** | **R^2^** | **inc *F*** |
| --- | --- | --- | --- | --- |
| 2 | 18.10 | 14.40 | 0.44 | NA |
| 3 | 13.58 | 18.92 | 0.58 | 232.24 |
| 4 | 12.13 | 20.37 | 0.63 | 83.74 |
| 5 | 10.78 | 21.72 | 0.67 | 87.41 |
| 6 | 10.22 | 22.28 | 0.69 | 37.85 |
| 7 | 9.97 | 22.53 | 0.69 | 17.46 |
| 8 | 9.19 | 23.31 | 0.72 | 59.03 |
| 9 | 8.63 | 23.87 | 0.73 | 44.77 |
| 10 | 8.42 | 24.08 | 0.74 | 17.25 |
| 11 | 8.27 | 24.23 | 0.75 | 12.34 |
| 12 | 7.74 | 24.77 | 0.76 | 48.11 |
| 13 | 7.02 | 25.48 | 0.78 | 69.89 |
| 14 | 6.52 | 25.98 | 0.80 | 53.01 |
| 15 | 6.35 | 26.15 | 0.80 | 18.64 |
| 16 | 6.15 | 26.36 | 0.81 | 22.65 |
| 17 | 5.80 | 26.70 | 0.82 | 40.54 |
| 18 | 5.77 | 26.73 | 0.82 | 3.31 |
| 19 | 5.48 | 27.02 | 0.83 | 36.94 |
| 20 | 5.26 | 27.24 | 0.84 | 28.37 |
| 21 | 5.06 | 27.44 | 0.84 | 26.30 |
| 22 | 4.99 | 27.51 | 0.85 | 9.53 |
| 23 | 4.82 | 27.68 | 0.85 | 25.00 |
| 24 | 4.75 | 27.75 | 0.85 | 8.70 |
| 25 | 4.65 | 27.85 | 0.86 | 15.03 |
| 26 | 4.57 | 27.93 | 0.86 | 12.62 |
| 27 | 4.40 | 28.10 | 0.86 | 25.36 |
| 28 | 4.29 | 28.21 | 0.87 | 17.95 |
| 29 | 4.25 | 28.25 | 0.87 | 6.43 |
| 30 | 4.13 | 28.37 | 0.87 | 19.08 |
